# Supplementary material for: Alterations in Plasma Lipid Profiles Associated with Melanoma and Therapy Resistance
Source: Int J Mol Sci. 2024 Jan 26;25(3):1558. doi: 10.3390/ijms25031558 (PMC10855791; doi:10.3390/ijms25031558)
Supplement: Supplementary file 1 [file ijms-25-01558-s001.zip › ijms-2822312-supplementary.pdf]

Supplementary Materials

Article

Alterations in plasma lipid profiles associate with melanoma and therapy resistance

Michele Dei Cas[1*], Chiara Maura Ciniselli[2*], Elisabetta Vergani[3*], Emilio Ciusani[4], Mariachiara Aloisi[3], Valeria Duroni[2], Paolo Verderio[2], Riccardo Ghidoni[1], Rita Paroni[1], Paola Perego[5], Giovanni Luca Beretta[5§], Laura Gatti[6*] and Monica Rodolfo[3*]

1 Clinical Biochemistry and Mass Spectrometry Laboratory, Health Sciences Department, Università degli Studi di Milano, Milan, Italy; michele.deicas@unimi.it; riccardo.ghidoni@unimi.it; rita.paroni@unimi.it

[2] Unit of Bioinformatics and Biostatistics, Department of Epidemiology and Data Science, Fondazione IRCCS Istituto Nazionale dei Tumori, Milan, Italy; chiara.ciniselli@istitutotumori.mi.it; paolo.verderio@istitutotumori.mi.it; valeria.duroni@istitutotumori.mi.it

[3] Unit of Translational Immunology, Department of Experimental Oncology, Fondazione IRCCS Istituto Nazionale dei Tumori di Milano, Milan, Italy; monica.rodolfo@istitutotumori.mi.it; vergani.eli@gmail.com; mariachiara.aloisi@hotmail.com

[4] Department of Diagnostic and Technology, Fondazione IRCCS Istituto Neurologico Carlo Besta, 20133 Milan, Italy; emilio.ciusani@istituto-besta.it

[5] Molecular Pharmacology Unit, Department of Experimental Oncology, Fondazione IRCCS Istituto Nazionale dei Tumori, 20133 Milan, Italy; paola.perego@istitutotumori.mi.it; giovanni.beretta@istitutotumori.mi.it

[6] Laboratory of Neurobiology and UCV, Neurology IX Unit, Fondazione IRCCS Istituto Neurologico Carlo Besta, 20133 Milan, Italy; laura.gatti@istituto-besta.it

[*] Authors contributing equally

§ Correspondence: giovanni.beretta@istitutotumori.mi.it; Tel.: +39-02-23903080

Table S1. Plasma levels of FASN, DHCR24, TG, CHOL, LDL and HDL: Results from univariate logistic regression models according to disease treatment response

	OR (95% CI)*	OR (95% CI)**
DHCR24 (pg/mL)	0.53 (0.19; 1.48)	0.52 (0.19; 1.47)
FASN (ng/mL)	1.54 (0.96; 2.47)	1.55 (0.96; 2.49)
TG (mg/dL)	0.89 (0.54; 1.47)	0.90 (0.55; 1.47)
Total CHOL (mg/dL)	1.11 (0.69; 1.79)	1.10 (0.68; 1.77)
LDL (mg/dL)	1.20 (0.74; 1.93)	1.19 (0.74; 1.91)
HDL (mg/dL)	0.98 (0.61; 1.59)	0.97 (0.60; 1.57)

*Odds ratio computed for a specific unit change for each variable equal to one SD; ** Odds ratio computed for a specific unit change for each variable equal to one SD and adjusted by age

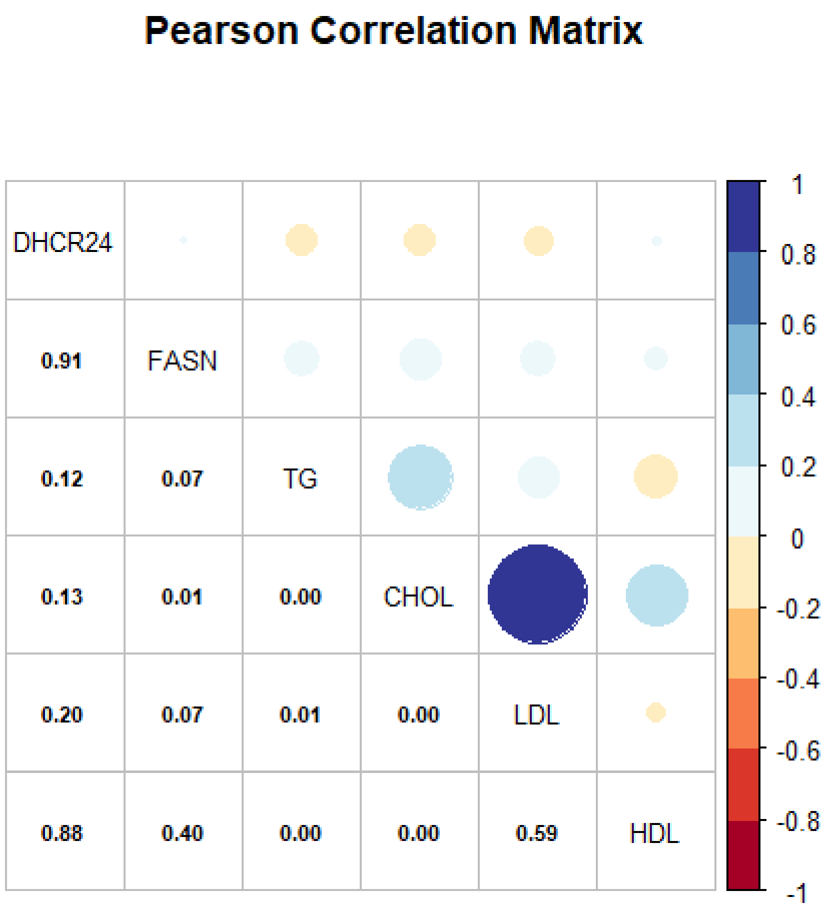

Figure S1. Correlogram summarizing relationships between FASN, DHCR24, CHOL, LDL and TG plasma levels as continuous variables. Pairwise relationships in terms of Pearson correlation coefficient r between continuous variables are shown. Colors indicate the direction of the correlation, with blue for positive and red from negative correlations, and the size of the bubble in the upper triangular matrix form indicate the corresponding magnitude. The p-values are indicated in the lower part of the matrix.
